# Gas-Sensing Performances of Metal Oxide Nanostructures for Detecting Dissolved Gases: A Mini Review

**DOI:** 10.3389/fchem.2020.00076

**Published:** 2020-02-21

**Authors:** Wei Guan, Na Tang, Kuang He, Xiaoying Hu, Mingshan Li, Kaiming Li

**Affiliations:** ^1^South China Institute of Environmental Sciences, Ministry of Ecological Environment, Guangzhou, China; ^2^The Key Laboratory of Water and Air Pollution Control of Guangdong Province, South China Institute of Environmental Sciences, Ministry of Environmental Protection (MEP), Guangzhou, China; ^3^Chongqing Solid Wastes Management Center, Chongqing, China

**Keywords:** metal oxides nanostructures, gas sensors, gas sensing mechanism, gas sensing performance, modification methods

## Abstract

Gas sensors have been wildly used in various fields related to people's lives. Gas sensor materials were the core factors that affected the performances of various gas sensors, and these have attracted much attention from scientific researchers due their high sensitivity, high selectivity, adjustable reliability, low cost, and other advantages. The preparation of nanostructures with a highly specific surface area was a useful method to improve the gas-sensing performance of a metal oxide semiconductor. Meanwhile, lots of research has focused on preparing nanostructures with a highly specific surface area. This paper has explored some fabricated sensors with high sensitivity, good selectivity, and long-term stability, which has also made them promising candidates for toxic gas detection. Besides, this paper has reviewed the development status of metal oxides used as gas sensors.

## Introduction

Gas sensors depends on converting gas into electrical signal output through chemical and physical effects in order to detect the composition and concentration of gas. Gas sensors are widely used in the fields of flammable detection, explosive detection, toxic and harmful gases detection, and environmental control (Zhou et al., 2019).

Recently, most of the gas sensors have been surface-controlled resistance sensors in which the sensitive materials of semiconductor resistance gas sensors are mainly concentrated in metal oxide semiconductors (Jeong et al., 2019). Due to a large number of free electrons in the conduction band and oxygen vacancies on the surface of the metal semiconductors, the material surface has strong adsorption characteristics and high reactivity and is changed under the action of surface gas. Therefore, the measurements can be made based on the electrical parameters (Rabee et al., 2019). Metal oxide materials have outstanding physical and chemical properties, are of low cost to produce, and have simple preparation methods (Wang et al., 2019). Therefore, they have been increasingly used in gas sensing.

Song et al. prepared hollow porous core-shell NiO nanotubes using a hydrothermal method. There were many micropores on the wall of a single nanotube with a specific surface area of 97.3 m^2^ g^−1^ (Song et al., 2011). Due to the large specific surface area, the sensitivity of the NiO gas sensor to 50 ppm ethanol gas was very high. It was believed that the hollow porous core-shell structure enabled ethanol molecules to diffuse and transport rapidly into the interior of the sensor, and so the material showed an excellent gas-sensing performance.

Ma et al. prepared hollow microtubules of In_2_O_3_ with degreased cotton as a soft biological template (Ma et al., 2019). The length of the hollow microtubule was 50–70 μm, the width was 5–6 μm, and the wall thickness was about 1 μm. The R_gas_/R_air_ value to 10 ppm Cl_2_ gas was 1,051 for the hollow microtubule sensor of In_2_O_3_, which was 25 times higher than that of In_2_O_3_ particles at 200°C. The existence of the hollow morphology of In_2_O_3_ with a highly specific surface area, rich oxygen vacancy, and narrow band gap were the reasons for the improving gas-sensing performance.

Wanit et al. prepared the ZnO nano-trees with a multistage branching structure through a hydrothermal growth method where numerous nano trees with dense distribution formed a vast nano forest (Wanit et al., 2012). This nano forest structure was suitable for dye-sensitized solar cells; it was similar to the role of the actual forest, which was used to convert solar energy into electricity. Obviously, this kind of nano forest structure had a very large specific surface area, which could be used significantly improve the gas-sensing performance of materials. Besides, the experiments indicated that this structure could greatly improve the light conversion efficiency and the overall light conversion efficiency was five times higher than that of dye-sensitized solar cells constructed by vertical ZnO nanowires. The improvement of light conversion efficiency was due to the large increase of specific surface area, which made the photosensitive dye adhesion area more extensive and could capture more sunlight. Moreover, the multi-level branch of the “nano tree” provided a direct conduction path for the charge and reduced the neutralization of the charge in the transmission process.

## Research Status of Metal Oxides for Gas Sensors

Gas sensors mainly include semiconductor gas sensors, electrochemical gas sensors, and contact combustion gas sensors, among which the most promising ones are the semiconductor gas sensors. With the application of a series of oxide semiconductors, including NiO, ZnO, SnO_2_, and CdO, the research into metal oxide for gas sensing is popular. The oxidation semiconductor refers to the one whose conductivity increases with the oxidation atmosphere, which belongs to the p-type semiconductor. The reduction semiconductor refers to the one whose conductivity increases with the reduction atmosphere, which belongs to the n-type semiconductor. The amphoteric semiconducting refers to the one whose conductivity type forms the p-type or n-type semiconductor with the oxygen partial pressure in the atmosphere. There are some methods to improve the gas-sensing performance, such as surface modification with organic molecules, perpetration of inorganic heterojunction sensitization, generation of hybrid structures with 1D or 2D materials, and oxygen vacancy modification (Zhang et al., 2019).

Adding a small amount of precious metals to semiconductor materials is an effective way to improve the gas-sensitive properties of materials. Shaver first found that the noble metal Pt had a significant enhancement for improving the sensitivity and selectivity of gas sensors, and that it also shortened the response time for WO_3_ oxide semiconductor gas sensors. In addition, adding a certain amount of rare earth metals to the material was also an effective way to improve the sensitivity and selectivity of the material.

Wang et al. prepared a series of polyaniline-TiO_2_ nanocomposites on the TiO_2_ surface by *in-situ* chemical oxidation polymerization of aniline (Wang et al., 2017). It was found that the polyaniline chain formed by TiO_2_ and polyaniline could increase the CO adsorption, which led to much more electrons transfer from CO to polyaniline. Following from polyaniline to TiO_2_ through a hydrogen bond, this resulted in promoting the photocatalytic oxidation of CO on the composite film sensor. This study provided a method for the development of a gas-sensitive material with organic-inorganic hybrid nanocomposites.

Nulhakim et al. prepared highly Ga-doped ZnO polycrystalline thin films deposited by radio-frequency magnetron sputtering for hydrogen gas sensing (Nulhakim et al., 2017). The relationship between the microstructure of preferred *c*-axis-oriented thin films and the hydrogen gas-sensing performance was introduced. It is found that the sensitivity of the sample to hydrogen increased slightly with the decrease of the microcrystalline size under the working temperature of 330°C. Moreover, the sensitivity was significantly improved by increasing the preferred orientation distribution. It was concluded that the c-axis orientation had a great influence on the sensitivity of the hydrogen gas sensor.

Amani et al. prepared a WO_3_ semiconductor gas sensor by electron beam evaporation technology, and the gas-sensing characteristics of the WO_3_ nano film activated by Pt and Au were studied (Amani et al., 2017). The results indicated that the existence of a Pt and Cu layer reduced the working temperature of the WO_3_ sensor; the WO_3_ nano thin film activation by the Pt layer significantly reduced the working temperature of the sensor.

Yang et al. synthesized ultralong MoO_3_ nanobelts with an average length of 200 μm and width of 200–400 nm by simple hydrothermal technology (Yang et al., 2016). The results indicated that the gas sensor prepared by an ultralong MoO_3_ nanobelt had a good gas-sensing performance for trimethylamine under the temperature of 240°C. The selectivity test of different reducing gases showed that the gas sensor had a better response to TMA than other gases, such as ethanol, ammonia, toluene, methanol, and acetone.

Zolghadr et al. conducted research on the effect of annealing temperatures at 600°C and 800°C on the gas-sensing properties of α-Fe_2_O_3_ (Zolghadr et al., 2016). The results showed that the gas sensitivity of α-Fe_2_O_3_ film annealed at 800°C was more stable, and the gas sensitivity of α-Fe_2_O_3_ to NO_2_ gas was higher than that of other gases.

Liu et al. prepared SnO_2_ thin films for gas sensors by ultrasonic spray pyrolysis technology and showed the effect of Cu doping amount on the gas-sensing performance (Liu et al., 2017). The results indicated that Cu doping could improve the gas sensitivity of SnO_2_ films, which was incorporated at the Cu/Sn ratio of 0–1.

**Figure 1:**
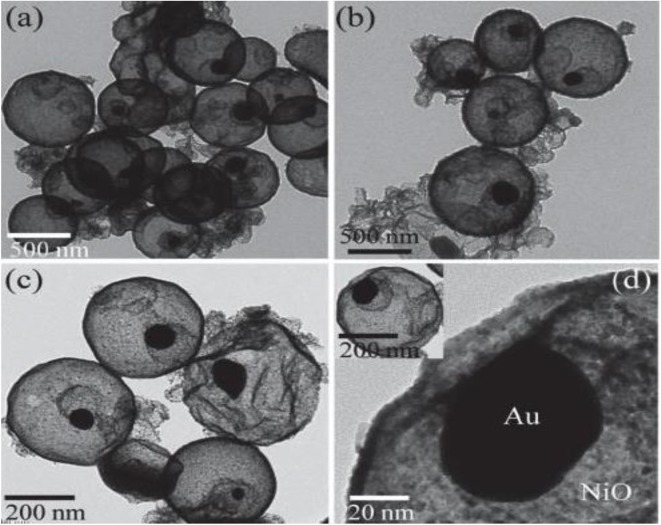
**(a–d)** TEM images of the Au/NiO core-shell structure. Reprinted with permission from Rai et al. (2014). Copyright (2014) Elsevier Science BV.

Rai et al. synthesized the Au/NiO core-shell structure with uniform dispersion by two-step hydrothermal method (Rai et al., 2014). As shown in Figure 1, Au existing alone in the NiO spherical shell showed an excellent gas-sensing performance for hydrogen sulfide gas. A wonderful rotten egg connection of gas-sensing material and gas was generated. The results indicated that the sensitivity to H_2_S gas for the NiO core-shell structure with Au as the core was nearly four times higher than that of the NiO sphere. Moreover, the sulfurization layer formed on the surface of Au particles adsorbed H_2_S gas; this reduced the reaction potential energy and made it easier for electrons to transfer from Au particles to the NiO shell and increased the resistance value.

**Figure 2:**
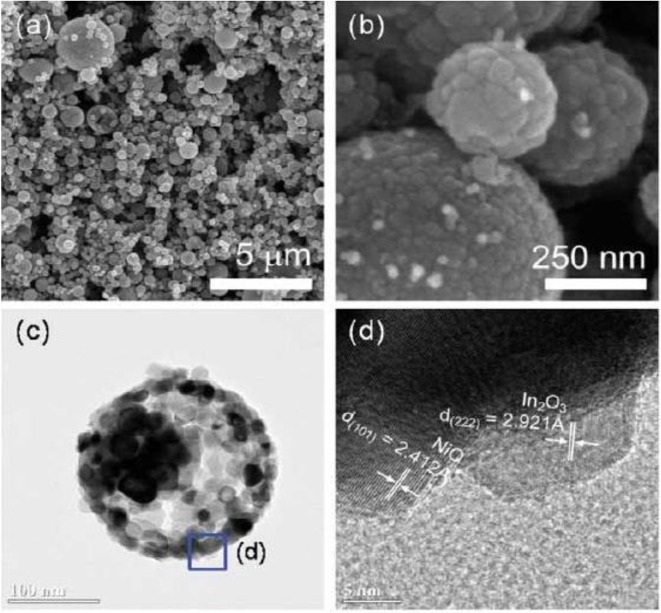
**(a,b)** SEM image of In_2_O_3_-Decorated NiO hollow nanostructures. **(c,d)** TEM image of In_2_O_3_-Decorated NiO hollow nanostructures. Reprinted with permission from Kim et al. (2014) Copyright 2014 American Chemical Society.

Kim et al. also synthesized the NiO hollow sphere nanostructure and used In_2_O_3_ to modify the NiO hollow sphere (Kim et al., 2014). The results indicated that the sensitivity to ethanol gas for In_2_O_3_-Decorated NiO hollow nanostructures was nearly five times higher than that of NiO sphere. The existence of n-type semiconductor of In_2_O_3_ reduced the accumulation of holes on the surface of NiO shell, as shown in Figure 2, which greatly reduced time for response and recovery to ethanol gas.

Finally, the gas-sensing mechanisms for metal oxides are the change in electrical signal caused by the gas. Gas-sensing mechanisms are included in two parts. One part explains the changes in electrical properties from a relatively microscopic perspective and includes mechanisms. The other part is relatively macroscopic, and it focus on the relationship between materials and gases (Ji et al., 2019).

## Conclusion

The research included some metal oxides used for gas sensing; some modification methods, such as metal oxide doping, metal/oxide heterojunction, metal oxide/metal oxide heterojunction, and metal oxide/conductive polymer heterojunction were introduced. The theoretical qualitative models of gas sensing include an atomic valence control model, double electric layer model, and grain boundary barrier model. Many researchers also put forward reasonable explanations based on the actual performance of the samples according to their specific research situation. The preparation of nanostructures with highly specific surface areas was a useful method to improve the gas-sensing performance.

## Author Contributions

WG and ML wrote the manuscript. All the authors read and approved the manuscript.

### Conflict of Interest

The authors declare that the research was conducted in the absence of any commercial or financial relationships that could be construed as a potential conflict of interest.
